# Supplementary material for: Factors influencing the outcomes of non-pharmacological interventions for managing fatigue across the lifespan of people living with musculoskeletal (MSK) conditions: a scoping review protocol
Source: BMJ Open. 2024 May 3;14(5):e082555. doi: 10.1136/bmjopen-2023-082555 (PMC11086445; doi:10.1136/bmjopen-2023-082555)
Supplement: Supplementary data [file bmjopen-2023-082555supp001.pdf]

Appendix 1: Search strategy overview

	Population	Exposure	Outcome
**Search terms**	musculoskeletal condition<br>musculoskeletal disease<br>inflammatory arthritis<br>rheumat*<br>osteoarthritis<br>fibromyalgia<br>juvenile idiopathic arthritis<br>juvenile chronic arthritis<br>spondyloarth*<br>systemic sclerosis<br>Sjogren's syndrome<br>lupus<br>psoriatic arthritis<br>spondylitis<br>scleroderma<br>*myositis<br>immune mediated necrotising<br>myopathy<br>anti synthetase syndrome<br>vasculitis<br>giant cell arteritis<br>Takayasu's arteritis<br>polyarteritis nodosa<br>granulomatosis with polyangiitis<br>microscopic angiitis<br>Behcet syndrome<br>Wegener syndrome<br>Churg-Strauss syndrome<br>low* back pain<br>Ehlers Danlos<br>hypermobility	non-pharmacological intervention<br>physical activity<br>exercis*<br>mindfulness<br>cognitive behavioural therapy<br>psycho-educational<br>psychosocial<br>education*<br>complementary<br>holistic therap*<br>nutrition<br>diet<br>massage<br>nurse-led<br>physiotherapy<br>occupational therapy<br>electro-physical modalities<br>thermotherapy<br>manual therapy<br>balneotherapy<br>tai chi<br>yoga<br>reflexology<br>aromatherapy<br>chiropractic<br>acupuncture<br>electroacupuncture<br>low level laser therapy<br>electric stimulation therapy<br>hyperthermia<br>walk*<br>pacing<br>hydrotherapy<br>activity diar*<br>swim*<br>dry needl*	fatigue
**Databases**	- EBSCO (AMED, CINAHLPlus, MEDLINE, PsycINFO)<br>- Ovid (Embase)<br>- Scopus<br>- Cochrane Database		
**Inclusion criteria**	- Primary research study<br>- Published in a peer reviewed journal<br>- Available in English language<br>- Participants have one or more chronic musculoskeletal conditions<br>- Participants experience fatigue at baseline<br>- Published during or after 2007<br>- Describes an intervention to manage MSK condition symptoms, with fatigue reduction as a primary or secondary outcome		

**Exclusion criteria**	- Reviews, protocols, opinion pieces, editorials, case reports, case series, observational cohort studies<br>- Pharmacological interventions<br>- No intervention is described<br>- Muscle fatigue rather than global fatigue is examined<br>- No data is available on factors associated with intervention success (theoretical mechanism of intervention OR characteristics of participants OR characteristics of clinicians delivering interventions)

Appendix 2: Amended JBI data extraction table

	**Study number**	**1**	**2**	**3**	**4**
**Study Information**	Author(s)				
	Title				
	Publication year				
	Country				
	Methodology				
	Sample size (participants, arms, control)				
	Condition(s) included				
	Main conclusion				
**Intervention**	Description of intervention				
	Hypothesis supporting intervention design and/or proposed mechanism of action				
	Characteristics of person(s) delivering intervention (e.g., profession, clinical skills, training to deliver intervention)				
**Participant details**	Age				
	Gender				
	Ethnicity				
	Recruitment setting (e.g., primary care, secondary care etc.)				
	Other participant characteristics (e.g., time since diagnosis, patient activation level, social context, optimised pharmacological management, comorbidities)				

Appendix 3: Proposed search strategy

3.1 EBSCO (AMED, CINAHLPlus, MEDLINE, PsycINFO)

S1 TITLE (“musculoskeletal condition” OR “musculoskeletal disease” OR “inflammatory arthritis” OR rheumat* OR osteoarthritis OR fibromyalgia OR “juvenile idiopathic arthritis” OR “juvenile chronic arthritis” OR spondyloarth* OR “systemic sclerosis” OR “Sjogren’s syndrome” OR lupus OR “psoriatic arthritis” OR spondylitis OR scleroderma OR *myositis OR “immune-mediated necrotising myopathy” OR “anti-synthetase syndrome” OR vasculitis OR “giant cell arteritis” OR “Takayasu’s arteritis” OR “polyarteritis nodosa” OR “granulomatosis with polyangiitis” OR “microscopic angiitis” OR “Behcet syndrome” OR “Wegener syndrome” OR “Churg-Strauss syndrome” OR “low* back pain” OR “Ehlers Danlos” OR hypermobility) OR ABSTRACT (“musculoskeletal condition” OR “musculoskeletal disease” OR “inflammatory arthritis” OR rheumat* OR osteoarthritis OR fibromyalgia OR “juvenile idiopathic arthritis” OR “juvenile chronic arthritis” OR spondyloarth* OR “systemic sclerosis” OR “Sjogren’s syndrome” OR lupus OR “psoriatic arthritis” OR spondylitis OR scleroderma OR *myositis OR “immune-mediated necrotising myopathy” OR “anti-synthetase syndrome” OR vasculitis OR “giant cell arteritis” OR “Takayasu’s arteritis” OR “polyarteritis nodosa” OR “granulomatosis with polyangiitis” OR “microscopic angiitis” OR “Behcet syndrome” OR “Wegener syndrome” OR “Churg-Strauss syndrome” OR “low* back pain” OR “Ehlers Danlos” OR hypermobility)

S2 TITLE (“non-pharmacological intervention” OR “physical activity” OR exercis* OR mindfulness OR “cognitive behavioural therapy” OR “psycho-educational” OR psychosocial OR education* OR complementary OR “holistic therap*” OR nutrition OR diet OR massage OR “nurse-led” OR physiotherapy OR “occupational therapy” OR “sports rehabilitation therapy” OR “electro-physical modalities” OR thermotherapy OR “manual therapy” OR balneotherapy OR “tai chi” OR yoga OR reflexology OR aromatherapy OR chiropractic OR acupuncture OR electroacupuncture OR “low level laser therapy” OR “electric stimulation therapy” OR hyperthermia OR walk* OR pacing OR hydrotherapy OR “activity diar*” OR swim* OR “dry needl*”) OR ABSTRACT (“non-pharmacological intervention” OR “physical activity” OR exercis* OR mindfulness OR “cognitive behavioural therapy” OR “psycho-educational” OR psychosocial OR education* OR complementary OR “holistic therap*” OR nutrition OR diet OR massage OR “nurse-led” OR physiotherapy OR “occupational therapy” OR “sports rehabilitation therapy” OR “electro-physical modalities” OR thermotherapy OR “manual therapy” OR balneotherapy OR “tai chi” OR yoga OR reflexology OR aromatherapy OR chiropractic OR acupuncture OR electroacupuncture OR “low level laser therapy” OR “electric stimulation therapy” OR hyperthermia OR walk* OR pacing OR hydrotherapy OR “activity diar*” OR swim* OR “dry needl*”)

S3 TITLE (fatigue) OR ABSTRACT (fatigue)

S4 S1 AND S2 AND S3

S5 Limit S5 to;

- Publication date during or after 2007
- English language
- Peer reviewed

3.2 Ovid (EMBASE)

#1 (musculoskeletal condition OR musculoskeletal disease OR inflammatory arthritis OR rheumat* OR osteoarthritis OR fibromyalgia OR juvenile idiopathic arthritis OR spondyloarth* OR systemic sclerosis OR Sjogrens syndrome OR lupus OR psoriatic arthritis OR spondylitis OR scleroderma OR myositis OR immune-mediated necrotising myopathy OR anti-synthetase syndrome OR vasculitis OR giant cell arteritis OR Takayasus arteritis OR polyarteritis nodosa OR granulomatosis with polyangiitis OR microscopic angiitis OR Behcet syndrome OR Wegener syndrome OR Churg-Strauss syndrome OR low* back pain OR Ehlers Danlos OR hypermobility).ti OR (musculoskeletal condition OR musculoskeletal disease OR inflammatory arthritis OR rheumat* OR osteoarthritis OR fibromyalgia OR juvenile idiopathic arthritis OR spondyloarth* OR systemic sclerosis OR Sjogrens syndrome OR lupus OR psoriatic arthritis OR spondylitis OR scleroderma OR myositis OR immune-mediated necrotising myopathy OR anti-synthetase syndrome OR vasculitis OR giant cell arteritis OR Takayasus arteritis OR polyarteritis nodosa OR granulomatosis with polyangiitis OR microscopic angiitis OR Behcet syndrome OR Wegener syndrome OR Churg-Strauss syndrome OR low* back pain OR Ehlers Danlos OR hypermobility).ab.

#2 Limit 1 to (English language and yr=”2007”)

#3 (fatigue).ti. or (fatigue).ab.

#4 Limit 2 to (English language and yr=”2007”)

#5 (non-pharmacological intervention OR physical activity or exercis* OR mindfulness OR cognitive behavioural therapy OR psycho-educational OR psychosocial OR education* OR complementary OR holistic therp* OR nutrition OR diet OR massage OR nurse-led OR physiotherapy OR occupational therapy OR sports rehabilitation therapy OR electro-physical modalities OR thermotherapy OR manual therapy OR balneotherapy OR tai chi OR yoga OR reflexology OR aromatherapy OR chiropractic OR acupuncture OR electroacupuncture OR low level laser therapy OR electric stimulation therapy OR hyperthermia OR walk* OR pacing OR hydrotherapy OR activity diar* OR swim* OR dry needl*).ti. OR (non-pharmacological intervention OR physical activity or exercis* OR mindfulness OR cognitive behavioural therapy OR psycho-educational OR psychosocial OR education* OR complementary OR holistic therp* OR nutrition OR diet OR massage OR nurse-led OR physiotherapy OR occupational therapy OR sports rehabilitation therapy OR electro-physical modalities OR thermotherapy OR manual therapy OR balneotherapy OR tai chi OR yoga OR reflexology OR aromatherapy OR chiropractic OR acupuncture OR electroacupuncture OR low level laser therapy OR electric stimulation therapy OR hyperthermia OR walk* OR pacing OR hydrotherapy OR activity diar* OR swim* OR dry needl*).ab.

#6 Limit 5 to (English language and yr=”2007”)

#7 2 and 4 and 6

3.3 SCOPUS

#1 (TITLE-ABS("non pharmacological intervention" OR "physical activity" OR exercis* OR mindfulness OR "cognitive behavioural therapy" OR psycho-educational OR psychosocial OR education* OR complementary OR “holistic therap*” OR nutrition OR diet OR massage OR "nurse-led” OR physiotherapy OR "occupational therapy" OR “sports rehabilitation therapy” OR "electro-physical modalities" OR thermotherapy OR "manual therapy" OR balneotherapy OR "tai chi" OR yoga OR reflexology OR aromatherapy OR chiropractic OR acupuncture OR electroacupuncture OR "low level laser therapy" OR "electric stimulation therapy" OR hyperthermia OR walk* OR pacing OR hydrotherapy OR “activity diar*” OR swim* OR “dry needl*”)

#2 Limit #1 to:

- Publication during or after 2007
- English language

#3 TITLE-ABS(fatigue)

#4 Limit #3 to:

- Publication during or after 2007
- English language

#5 TITLE-ABS(“musculoskeletal condition” OR “musculoskeletal disease” OR “inflammatory arthritis” OR rheumat* OR osteoarthritis OR fibromyalgia OR “juvenile idiopathic arthritis” OR spondyloarth* OR “systemic sclerosis” OR “Sjogrens syndrome” OR lupus OR “psoriatic arthritis” OR spondylitis OR scleroderma OR *myositis OR “immune-mediated necrotising myopathy” OR “anti-synthetase syndrome” OR vasculitis OR “giant cell arteritis” OR “Takayasus arteritis” OR “polyarteritis nodosa” OR “granulomatosis polyangiitis” OR “microscopic angiitis” OR “Behcet syndrome” OR “Wegener syndrome” OR “Churg-Strauss syndrome” OR “low* back pain” OR “Ehlers Danlos” OR hypermobility)

#6 Limit #5 to:

- Publication during or after 2007
- English language

#7 #2 AND #4 AND #6

3.4 COCHRANE LIBRARY DATABASE

ID Search terms Results

#1 ("non pharmacological intervention" OR "physical activity" OR exercis* OR mindfulness OR "cognitive behavioural therapy" OR psycho-educational OR psychosocial OR education* OR complementary OR “holistic therap*” OR nutrition OR diet OR massage OR "nurse-led” OR physiotherapy OR "occupational therapy" OR “sports rehabilitation therapy” OR "electro-physical modalities" OR thermotherapy OR "manual therapy" OR balneotherapy OR "tai chi" OR yoga OR reflexology OR aromatherapy OR chiropractic OR acupuncture OR electroacupuncture OR "low level laser therapy" OR "electric stimulation therapy" OR hyperthermia OR walk* OR pacing OR hydrotherapy OR “activity diar*” OR swim* OR “dry needl*”):ti,ab

#2 (fatigue):ti,ab

#3 (“musculoskeletal condition” OR “musculoskeletal disease” OR “inflammatory arthritis” OR rheumat* OR osteoarthritis OR fibromyalgia OR “juvenile idiopathic arthritis” OR spondyloarth* OR “systemic sclerosis” OR “Sjogren’s syndrome” OR lupus OR “psoriatic arthritis” OR spondylitis OR scleroderma OR *myositis OR “immune-mediated necrotising myopathy” OR “anti-synthetase syndrome” OR vasculitis OR “giant cell arteritis” OR “Takayasu’s arteritis” OR “polyarteritis nodosa” OR “granulomatosis with polyangiitis” OR “microscopic angiitis” OR “Behcet syndrome” OR “Wegener syndrome” OR “Churg-Strauss syndrome” OR “low* back pain” OR “Ehlers Danlos” OR hypermobility):ti,ab

#4 #1 AND #2 AND #3

#5 Limit to:

- Publication date from Jan 2007 to Oct 2023
- English language
