# Supplementary material for: Factors influencing the outcomes of non-pharmacological interventions for managing fatigue across the lifespan of people living with musculoskeletal (MSK) conditions: a scoping review protocol
Source: BMJ Open. 2024 May 3;14(5):e082555. doi: 10.1136/bmjopen-2023-082555 (PMC11086445; doi:10.1136/bmjopen-2023-082555)
Supplement: Author's manuscript [file bmjopen-2023-082555.draft_revisions.pdf]

BMJ Open is committed to open peer review. As part of this commitment we make the peer review history of every article we publish publicly available.

When an article is published we post the peer reviewers' comments and the authors' responses online. We also post the versions of the paper that were used during peer review. These are the versions that the peer review comments apply to.

The versions of the paper that follow are the versions that were submitted during the peer review process. They are not the versions of record or the final published versions. They should not be cited or distributed as the published version of this manuscript.

BMJ Open is an open access journal and the full, final, typeset and author-corrected version of record of the manuscript is available on our site with no access controls, subscription charges or pay-per-view fees (http://bmjopen.bmj.com).

If you have any questions on BMJ Open's open peer review process please email info.bmjopen@bmj.com

BMJ Open

Factors influencing the outcomes of non-pharmacological interventions for managing fatigue across the lifespan of people living with musculoskeletal (MSK) conditions: a scoping review protocol

Journal:	*BMJ Open*
Manuscript ID	bmjopen-2023-082555
Article Type:	Protocol
Date Submitted by the Author:	27-Nov-2023
Complete List of Authors:	Fishpool, Katie; University of the West of England, School of Health and Social Wellbeing; University Hospitals Bristol and Weston NHS Foundation Trust, Academic Rheumatology<br>Young, George; University of the West of England<br>Ciurtin, C; University College London,<br>Cramp, Fiona; University of the West of England<br>Erhieyovwe, Emmanuel; Hywel Dda University Health Board<br>Farisogullari, Bayram; University College London<br>Macfarlane, Gary ; University of Aberdeen Aberdeen Centre for Arthritis and Musculoskeletal Health<br>Machado, Pedro M.; University College London, Centre for Rheumatology; University College London, Neuromuscular Diseases<br>Pearson, J; University of the West of England Bristol<br>Santos, Eduardo ; Hospitals of Coimbra University<br>Dures, Emma; University of the West of England; University Hospitals Bristol and Weston NHS Foundation Trust
Keywords:	Fatigue, Musculoskeletal disorders < ORTHOPAEDIC & TRAUMA SURGERY, RHEUMATOLOGY, Paediatric rheumatology < PAEDIATRICS

SCHOLARONE™
Manuscripts

Factors influencing the outcomes of non-pharmacological interventions for managing fatigue across the lifespan of people living with musculoskeletal (MSK) conditions: a scoping review protocol

Word count: 2278

Katie Fishpool[1,2], George Young[1], Coziana Ciurtin[3], Fiona Cramp[1], Emmanuel Erhieyovwe[4], Bayram Farisogullari[5], Gary Macfarlane[6], Pedro M. Machado[5,7], Jen Pearson[1], Eduardo Santos[8], Emma Dures[1,2]

Corresponding author: Katie Fishpool katie.fishpool@uwe.ac.uk

[1] College of Health and Social Wellbeing, College of Health, Science and Society, University of the West of England, Bristol, UK

[2] Academic Rheumatology, University Hospitals Bristol and Weston NHS Foundation Trust, UK

[3] Centre for Adolescent Rheumatology, Division of Medicine, UCL Queen Square Institute of Neurology, University College London, UK

[4] General Internal Medicine, Withybush General Hospital Haverfordwest, Hywel Dda University Health Board, UK

[5] Department of Neuromuscular Diseases, University College London, UK

[6] Aberdeen Centre for Arthritis and Musculoskeletal Health (Epidemiology Group), University of Aberdeen, UK

[7] Centre for Rheumatology, Division of Medicine, University College London, UK

[8] Polytechnic University of Viseu, School of Health, Portugal; Health Sciences Research Unit: Nursing (UICISA: E); Portugal Centre for Evidence Based Practice (PCEBP): a JBI Centre of Excellence, Portugal

ABSTRACT

Introduction

Fatigue is an important and distressing symptom for many people living with chronic musculoskeletal (MSK) conditions. Many non-pharmacological interventions have been investigated in recent years and some have been demonstrated to be effective in reducing fatigue and fatigue impact, however there is limited guidance for clinicians to follow regarding the most appropriate management options. The objective of this scoping review is to understand and map the extent of evidence in relation to the impact of non-pharmacological interventions on MSK condition-related fatigue across the lifespan.

Methods and analysis

This scoping review will include evidence relating to people of all ages living with chronic MSK conditions who have been offered a non-pharmacological intervention with either the intention or effect of reducing fatigue and its impact. Databases including AMED, PsycINFO, CINAHLPlus, MEDLINE, EMBASE and Scopus will be searched for peer reviewed primary research studies published after 1st January 2007 in English language. These findings will be used to identify factors associated with successful interventions and to map gaps in knowledge.

Ethics and dissemination

For peer review only

Ethical approval was not required for this review. Findings will be disseminated by journal publication, conference presentation and by communicating with relevant healthcare and charity organisations.

Keywords: chronic musculoskeletal condition, fatigue, lifespan, non-pharmacological intervention

Article Summary

Strengths and limitations of this study

- Patient and Public Involvement and Engagement (PPIE) workshops at key time points will ensure that the protocol, review findings and subsequent discussion are relevant to stakeholders and reflect lived experience of MSK-fatigue
- All studies will be reviewed, and data extraction checked by a minimum of two researchers
- The effectiveness of specific interventions and methodological quality of included studies is not covered in this scoping review
- Only evidence available in English will be reviewed

INTRODUCTION

Musculoskeletal (MSK) conditions include inflammatory and non-inflammatory conditions such as connective tissue diseases, inflammatory arthritis and osteoarthritis, back and neck pain, and fibromyalgia which affect the muscles, bones, joints and connective tissue (1,2). More than 10 million people in the UK and 1.7 billion people globally currently live with an MSK condition (1,2). Prevalence increases with age but these conditions are encountered across the lifespan, with approximately 234,000 children in England and Scotland living with a long term MSK condition (2).

Fatigue has been identified by people living with chronic MSK conditions as a priority symptom which has a significant impact on quality of life (2–8). Pharmacological treatments are not licensed for the management of fatigue without concurrent disease activity, so the focus in clinical practice has been on non-pharmacological options (9,10). This has been mirrored in healthcare research and recent systematic reviews have examined the strength of the evidence supporting a range of non-pharmacological interventions in different patient groups (11–15). Non-pharmacological interventions are any non-chemical or biological interventions that are theoretically based and empirically proven, or that have a logical rationale which is possible to prove by empirical study (11,16).

The European Alliance of Associations for Rheumatology (EULAR) recently funded a taskforce to examine the effectiveness of non-pharmacological interventions for fatigue in inflammatory rheumatic conditions and the resulting systematic review found strong evidence that some interventions are effective (14). This informed the EULAR recommendations for the management of fatigue (17) and highlighted a need to better understand contextual factors and the mechanisms by which interventions are effective (7). There is currently no comprehensive understanding of the factors which influence the success of an intervention. The impact of this is a lack of evidence to support decision making in how to design, offer and deliver interventions in the most effective way, tailored to a range of patients and at the optimal time. Current clinical guidelines for the management of common MSK conditions recognise fatigue as an important symptom but do not

For peer review only - http://bmjopen.bmj.com/site/about/guidelines.xhtml

make any recommendations for how it can be addressed directly (18–20), hindering implementation of the evidence.

The clinical pathway for management of MSK conditions in the UK differs depending on primary diagnosis, with suspected inflammatory conditions being referred to specialist secondary care settings and osteoarthritis and fibromyalgia being managed predominantly through primary care (20–22). The impact of this is that the experience of patients with MSK fatigue and the profession and skills of the clinicians who provide their care may be quite different.

Review aims

The aim of this scoping review is to generate evidence for health professionals and educators to design or adapt tailored MSK-fatigue support. The objectives of this review are to identify evidence for existing interventions for MSK-fatigue across the life course and to explore the theoretical basis for the interventions. To explore the comprehensive nature of the existing evidence, the clinical and demographic characteristics of the participants as well as to capture the training/skills of those who deliver the interventions where this information is available. As the intention is to create an overview of the current knowledge and to highlight gaps in the existing literature rather than to assess the effectiveness of specific interventions, a scoping review is the most appropriate methodological approach (23,24).

METHODS AND ANALYSIS

Study design

Recent and ongoing studies on the topic of fatigue support the need for this scoping review and have been used to inform its design. A previous scoping review exploring fatigue in patients with rheumatic and MSK conditions (25) reported on the efficacy of interventions and also considered determinants associated with fatigue. A systematic review assessed the quality of evidence available to support non-pharmacological interventions to reduce fatigue in patients with inflammatory rheumatic conditions (14). The National Institute for Health Research (NIHR) have recently awarded funding for the project “Effectiveness of Interventions For FatiguE in Long term conditions” (EIFFEL) (26). Study protocols have been registered on the PROSPERO database which state that the team will be conducting two systematic reviews; one to assess the effectiveness of interventions for fatigue in long term conditions (27) and the other to explore the acceptability of interventions for fatigue (28). There is potential for duplication of effort when considering acceptability of interventions, as the qualitative data may explore contextual factors and some musculoskeletal conditions are also included in their searches. However, the proposed methodology of this study is a scoping review which allows for a broader view of contextual factors across the lifespan of all chronic MSK conditions and will be gathered from multiple study designs, which is appropriate for the aims of the review to map existing knowledge. Contact has been made with the EIFFEL team to reduce risk of duplication and share knowledge.

The review has been designed in accordance with the Joanna Briggs Institute (JBI) methodology for scoping reviews (29) and will be reported using the PRISMA-ScR extension guidelines for scoping reviews (30).

Identifying relevant studies

For peer review only

A preliminary search of MEDLINE in August 2023 identified the current scope and scale of the evidence base related to the scoping review. The search strategy (Appendix 1) was then developed with support from a specialist subject librarian and reviewed by stakeholders in a PPIE workshop.

The following electronic databases will be searched for research published in peer-reviewed journals from January 2007 onwards; AMED, MEDLINE, PsycINFO, CINAHLPlus (EBSCO platform), EMBASE (Ovid platform), SCOPUS and Cochrane Database. This date was chosen to correspond with the Outcome Measures in Rheumatology 8th meeting (OMERACT 8) which endorsed fatigue as an addition to the 'core set' of outcome measures for all subsequent studies, highlighting the importance of investigating this symptom (31). There will be no restrictions on the age of participants, allowing interventions that have been used throughout the life course and highlighting any gaps in provision. A search for unpublished studies will not be conducted due to limitations of time to complete this review. The reference lists from included studies will be hand searched to check for any other relevant papers not captured in the database searching. Only evidence available in English will be reviewed and studies in other languages will be excluded due to time. All studies will be uploaded to the review software system Covidence (32).

Selection of studies

A minimum of two independent reviewers will screen the titles and abstracts of all identified studies against the stated inclusion and exclusion criteria (Table 1). Regular meetings will be held by the team throughout the title and abstract screening process to aid understanding and reduce disagreements (33). Papers that proceed to the full text stage of screening will also be reviewed by two or more independent reviewers who will document the main reason any excluded papers do not meet the inclusion criteria. Any differences in opinion between the reviewers will be resolved by discussing the papers, with an additional independent reviewer to support mediation, as required. Any reasons for exclusion of full-text papers that do not meet the inclusion criteria will be recorded and reported in the scoping review.

Table 1: Inclusion and exclusion criteria

**Inclusion criteria**	**Exclusion criteria**
- Primary research study<br>- Published in a peer reviewed journal<br>- Available in English language<br>- Participants have one or more chronic MSK conditions<br>- Participants experience fatigue at baseline<br>- Published during or after 2007<br>- Describes a non-pharmacological intervention to manage MSK condition symptoms, with fatigue reduction as a primary or secondary outcome	- Reviews, protocols, opinion pieces, editorials, case series, case reports, observational cohort studies<br>- Pharmacological interventions<br>- No intervention is described<br>- Muscle fatigue rather than global fatigue is examined<br>- No data is available on factors associated with intervention success (theoretical mechanism of intervention OR characteristics of participants OR characteristics of clinicians delivering interventions)

All primary research methodologies will be considered, including experimental and quasi-experimental study designs, before and after studies and interrupted timeseries studies, analytical observational studies, case-control studies and analytical cross-sectional studies, and descriptive

For peer review only - http://bmjopen.bmj.com/site/about/guidelines.xhtml

observational study designs. Qualitative studies will also be considered including, for example, phenomenology, grounded theory, ethnography, qualitative description, and action research.

Data extraction

Data from included papers will be extracted by one reviewer and checked by another using an adapted version of the JBI template for data extraction (Appendix 2). This template captures information about study participants, methods, and findings relevant to the research question (34) and has been amended to extract additional data on contextual factors including clinical characteristics, information on clinicians delivering interventions and the hypothesis behind the design of the intervention. Any disagreements that may arise between the reviewers will be resolved by consensus, with an additional reviewer to support mediation. If necessary, the authors of the included papers will be contacted for further information or data clarification.

Data reporting and analysis

Findings will be presented in a PRISMA (Preferred Reporting Items for Systematic Reviews and Meta Analyses) flow diagram (35) to demonstrate the number of articles identified and their sources, with reasons for exclusion at full text screening summarised. All included studies will be summarised in tabular format. Further figures will be used to illustrate a map of the existing literature and any gaps highlighted by the review. Data analysis is likely to be narrative due to the broad range of study types being included. This may be further refined for use during the review process.

Patient and public involvement statement

A patient and public involvement and engagement (PPIE) workshop was held in October 2023 during the design stage of the search strategy and review protocol. Stakeholders attending the workshop included patients living with one or more MSK conditions and clinicians from a range of professions who support patients that experience MSK related fatigue. The discussion focussed on people's experiences of offering or being offered support to manage their fatigue and asking for comments on the proposed review. This highlighted additional intervention types and pathways that were subsequently included in the search terms (Appendix 3). It also confirmed our understanding that fatigue is a significant issue and that an overview of potential interventions and management techniques would be welcomed by patients and clinicians.

Further PPIE events are planned at key points during the project to ensure the validity of the final review. Workshops that focus on the support of adults and of children and young people will be held to discuss the initial findings following data extraction with the aim of highlighting key themes and gaps in knowledge. A further pair of workshops will be arranged following synthesis of the findings to ensure the validity of the review, discuss priorities for future research and to promote dissemination of the findings through appropriate groups. The outcome of these events and how they influence the scoping review process will be reported in the final review following the guidelines for the GRIPP2 short form reporting checklist (36), which is a tool designed to improve the reporting of public and patient involvement in research.

ETHICS AND DISSEMINATION

Ethical approval is not required for this scoping review. The findings of this review will be disseminated via relevant peer-reviewed journals, conference presentations and through sharing findings with relevant charities and health professionals.

For peer review only

AUTHOR CONTRIBUTIONS

Project funding application by ED with support from FC, JP, GM, PM, CC, BF and ES. Drafting of protocol and search strategy by KF in discussion with ED and GY, reviewed by EE, FC, JP, GM, PM, CC, BF and ES.

FUNDING STATEMENT

This scoping review protocol is part of the project "MusculoskelEtal faTigue acRoss the lIfe CourSe: understanding what helps and mapping what is missing (METRICS)" (reference 23140), which is jointly funded by Versus Arthritis and The Kennedy Trust.

COMPETING INTERESTS STATEMENT

PMM has received consulting/speaker's fees from Abbvie, BMS, Celgene, Eli Lilly, Janssen, MSD, Novartis, Orphazyme, Pfizer, Roche and UCB, all unrelated to this project. There are no competing interests in this project.

ACKNOWLEDGEMENTS

The development of this scoping review search strategy was supported by Specialist Subject Librarian Pauline Shaw from the University of the West of England library. The review team is also grateful for the contributions of the stakeholders who attended the Patient and Public Involvement and Engagement workshop.

For peer review only

References

1. World Health Organisation (WHO). Musculoskeletal health. World Health Organisations News Room Fact Sheets 2022. Available from: https://www.who.int/news-room/fact-sheets/detail/musculoskeletal-conditions. (accessed 11 Oct 2023)

2. Versus Arthritis. The State of Musculoskeletal Health. Available from: https://www.versusarthritis.org/about-arthritis/data-and-statistics/the-state-of-musculoskeletal-health/ (accessed 11 Oct 2023)

3. Steunebrink LMM, Oude Voshaar MAH, Taal E, et al. Determinants of Perceived Health Nonimprovement in Early Rheumatoid Arthritis Patients With Favorable Treatment Outcomes. Arthritis Care Res. 2018 Apr;70(4):510–5.

4. Van Tuyl LHD, Sadlonova M, Hewlett S, et al. The patient perspective on absence of disease activity in rheumatoid arthritis: a survey to identify key domains of patient-perceived remission. Ann Rheum Dis. 2017 May;76(5):855–61.

5. Dean LE, Macfarlane GJ, Jones GT. Five Potentially Modifiable Factors Predict Poor Quality of Life in Ankylosing Spondylitis: Results from the Scotland Registry for Ankylosing Spondylitis. J Rheumatol. 2018 Jan;45(1):62–9.

6. Primdahl J, Hegelund A, Lorenzen AG, et al. The Experience of people with rheumatoid arthritis living with fatigue: a qualitative metasynthesis. BMJ Open. 2019 Mar;9(3):e024338.

7. Kant-Smits K, Van Brussel M, Nijhof S, et al. Reducing fatigue in pediatric rheumatic conditions: a systematic review. Pediatr Rheumatol. 2021 Jul 8;19(1):111.

8. Tollisen A, Selvaag AM, Aasland A, et al. Personally Generated Quality of Life Outcomes in Adults With Juvenile Idiopathic Arthritis. J Rheumatol. 2022 Oct 1;49(10):1138–45.

9. Druce KL, Jones GT, Macfarlane GJ, et al. Determining Pathways to Improvements in Fatigue in Rheumatoid Arthritis: Results From the British Society for Rheumatology Biologics Register for Rheumatoid Arthritis. Arthritis Rheumatol. 2015;67(9):2303–10.

10. Salmon VE, Hewlett S, Walsh NE, et al. Developing a group intervention to manage fatigue in rheumatoid arthritis through modifying physical activity. BMC Musculoskelet Disord. 2019 May 4;20(1):194.

11. Cramp F, Hewlett S, Almeida C, et al. Non-pharmacological interventions for fatigue in rheumatoid arthritis. Cochrane Database Syst Rev [Internet]. 2013. Available from: https://www.readcube.com/articles/10.1002%2F14651858.cd008322.pub2 (accessed 11 Oct 2023)

12. Bidonde J, Busch AJ, Schachter CL, et al. Mixed exercise training for adults with fibromyalgia. Cochrane Database Syst Rev [Internet]. 2019. Available from: https://www.cochranelibrary.com/cdsr/doi/10.1002/14651858.CD013340/full?highlightAbstract=fatigu%7Cfatigue (accessed 11 Oct 2023)

13. Regnaux JP, Davergne T, Palazzo C,et al . Exercise programmes for ankylosing spondylitis. Cochrane Database Syst Rev [Internet]. 2019 [cited 2023 Sep 11];(10). Available from: https://www.cochranelibrary.com/cdsr/doi/10.1002/14651858.CD011321.pub2/full (accessed 11 Oct 2023)

For peer review only

14. Santos EJF, Farisogullari B, Dures E, et al. EULAR taskforce on recommendations for the management of fatigue in people with inflammatory rheumatic diseases. Efficacy of non-pharmacological interventions: a systematic review informing the 2023 EULAR recommendations for the management of fatigue in people with inflammatory rheumatic and musculoskeletal diseases. RMD Open. 2023 Aug;9(3):e003350.

15. Frade S, O'Neill S, Greene D,et al. Exercise as adjunctive therapy for systemic lupus erythematosus. Cochrane Database Syst Rev [Internet]. 2023 Available from: https://www.cochranelibrary.com/cdsr/doi/10.1002/14651858.CD014816.pub2/full?highlightAbstract=fatigu%7Cfatigue (accessed 11 Oct 2023)

16. Olazarán J, Reisberg B, Clare L, et al. Nonpharmacological Therapies in Alzheimer's Disease: A Systematic Review of Efficacy. Dement Geriatr Cogn Disord. 2010;30(2):161–78.

17. Dures E, Farisogullari B, Santos E, et al. Pos0370 2023 Eular Recommendations for the Management of Fatigue in People with Inflammatory Rheumatic and Musculoskeletal Diseases. Ann Rheum Dis. 2023 Jun 1;82(Suppl 1):437–8.

18. Recommendations | Rheumatoid arthritis in adults: management | Guidance | NICE [Internet]. NICE; 2018. Available from: https://www.nice.org.uk/guidance/ng100/chapter/Recommendations (accessed 11 Oct 2023)

19. Recommendations | Spondyloarthritis in over 16s: diagnosis and management | Guidance | NICE [Internet]. NICE; 2017. Available from: https://www.nice.org.uk/guidance/NG65/chapter/Recommendations#recognition-and-referral-in-non-specialist-care-settings (accessed 11 Oct 2023)

20. Recommendations | Chronic pain (primary and secondary) in over 16s: assessment of all chronic pain and management of chronic primary pain | Guidance | NICE [Internet]. NICE; 2021, Available from: https://www.nice.org.uk/guidance/ng193/chapter/Recommendations#managing-chronic-primary-pain (accessed 11 Oct 2023)

21. NICE. Management of inflammatory arthritis | NICE impact arthritis | Reviewing the impact of our guidance | Measuring the use of NICE guidance | Into practice | What we do | About. Available from: https://www.nice.org.uk/about/what-we-do/into-practice/measuring-the-use-of-nice-guidance/impact-of-our-guidance/nice-impact-arthritis/management-of-inflammatory-arthritis (accessed 11 Oct 2023)

22. RCP London [Internet]. 2022. The diagnosis of fibromyalgia syndrome. Available from: https://www.rcplondon.ac.uk/guidelines-policy/diagnosis-fibromyalgia-syndrome (accessed 10 Oct 2023)

23. Peters MDJ, Marnie C, Tricco AC, et al. Updated methodological guidance for the conduct of scoping reviews. JBI Evid Implement. 2021 Mar;19(1):3–10.

24. Munn Z, Peters MDJ, Stern C, et al. Systematic review or scoping review? Guidance for authors when choosing between a systematic or scoping review approach. BMC Med Res Methodol. 2018 Nov 19;18(1):143.

25. Beckers E, Hermans K, Tubergen AV, et al. Fatigue in patients with rheumatic and musculoskeletal diseases: a scoping review on definitions, measurement instruments, determinants, consequences and interventions. RMD Open. 2023 Aug 1;9(3):e003056.

For peer review only

26. Effectiveness of Interventions For Fatigue in Long term conditions (EIFFEL) - NIHR Funding and Awards [Internet]. Available from: https://fundingawards.nihr.ac.uk/award/NIHR154660 (accessed 10 Oct 2023)

27. Leaviss J, Burton C, Booth A, et al. (Protocol) Effectivenesss of Interventions For Fatigue in Long term conditions (EIFFEL) [Internet]. 2023. Available from: https://www.crd.york.ac.uk/PROSPERO/display_record.php?RecordID=440141(accessed 11 Oct 2023)

28. Leaviss J, Booth A, Burton C, et al. (Protocol) Acceptability of Interventions For Fatigue in Long term conditions (EIFFEL) [Internet]. 2023. Available from: https://www.crd.york.ac.uk/PROSPERO/display_record.php?RecordID=459361(accessed 16 Oct 2023)

29. Peters MDJ, Godfrey C, McInerney P, et al. Chapter 11: Scoping reviews. In: JBI Manual for Evidence Synthesis [Internet]. JBI; 2020. Available from: https://synthesismanual.jbi.global (accessed 5 Sep 2023)

30. Tricco AC, Lillie E, Zarin W, et al. PRISMA Extension for Scoping Reviews (PRISMA-ScR): Checklist and Explanation. Ann Intern Med. 2018 Oct 2;169(7):467–73.

31. Kirwan JR, Minnock P, Adebajo A, et al. Patient perspective: fatigue as a recommended patient centered outcome measure in rheumatoid arthritis. J Rheumatol. 2007 May 1;34(5):1174–7.

32. Covidence [Internet]. Melbourne, Australia: Veritas Health Innovation; (Covidence systematic review software). Available from: www.covidence.org (accessed 20 Sep 2023)

33. Polanin JR, Pigott TD, Espelage DL, et al. Best practice guidelines for abstract screening large-evidence systematic reviews and meta-analyses. Res Synth Methods. 2019;10(3):330–42.

34. Appendix 11.1 JBI template source of evidence details, characteristics and results extraction instrument - JBI Manual for Evidence Synthesis - JBI Global Wiki [Internet]. Available from: https://jbi-global-wiki.refined.site/space/MANUAL/4687579/Appendix+11.1+JBI+template+source+of+evidence+details%2C+characteristics+and+results+extraction+instrument(accessed 16 Oct 2023)

35. PRISMA [Internet]. Available from: http://prisma-statement.org/PRISMAStatement/FlowDiagram(accessed 16 Oct 2023)

36. Staniszewska S, Brett J, Simera I, et al. GRIPP2 reporting checklists: tools to improve reporting of patient and public involvement in research. BMJ. 2017 Aug 2;358:j3453.

For peer review only

Appendix 1: Search strategy overview

	Population	Exposure	Outcome
**Search terms**	musculoskeletal condition<br>musculoskeletal disease<br>inflammatory arthritis<br>rheumat*<br>osteoarthritis<br>fibromyalgia<br>juvenile idiopathic arthritis<br>juvenile chronic arthritis<br>spondyloarth*<br>systemic sclerosis<br>Sjogren's syndrome<br>lupus<br>psoriatic arthritis<br>spondylitis<br>scleroderma<br>*myositis<br>immune mediated necrotising<br>myopathy<br>anti synthetase syndrome<br>vasculitis<br>giant cell arteritis<br>Takayasu's arteritis<br>polyarteritis nodosa<br>granulomatosis with polyangiitis<br>microscopic angiitis<br>Behcet syndrome<br>Wegener syndrome<br>Churg-Strauss syndrome<br>low* back pain<br>Ehlers Danlos<br>hypermobility	non-pharmacological intervention<br>physical activity<br>exercis*<br>mindfulness<br>cognitive behavioural therapy<br>psycho-educational<br>psychosocial<br>education*<br>complementary<br>holistic therap*<br>nutrition<br>diet<br>massage<br>nurse-led<br>physiotherapy<br>occupational therapy<br>electro-physical modalities<br>thermotherapy<br>manual therapy<br>balneotherapy<br>tai chi<br>yoga<br>reflexology<br>aromatherapy<br>chiropractic<br>acupuncture<br>electroacupuncture<br>low level laser therapy<br>electric stimulation therapy<br>hyperthermia<br>walk*<br>pacing<br>hydrotherapy<br>activity diar*<br>swim*<br>dry needl*	fatigue
**Databases**	- EBSCO (AMED, CINAHLPlus, MEDLINE, PsycINFO)<br>- Ovid (Embase)<br>- Scopus<br>- Cochrane Database		
**Inclusion criteria**	- Primary research study<br>- Published in a peer reviewed journal<br>- Available in English language<br>- Participants have one or more chronic musculoskeletal conditions<br>- Participants experience fatigue at baseline<br>- Published during or after 2007<br>- Describes an intervention to manage MSK condition symptoms, with fatigue reduction as a primary or secondary outcome		

For peer review only

**Exclusion criteria**	- Reviews, protocols, opinion pieces, editorials, case reports, case series, observational cohort studies<br>- Pharmacological interventions<br>- No intervention is described<br>- Muscle fatigue rather than global fatigue is examined<br>- No data is available on factors associated with intervention success (theoretical mechanism of intervention OR characteristics of participants OR characteristics of clinicians delivering interventions)

For peer review only

Appendix 2: Amended JBI data extraction table

	**Study number**	**1**	**2**	**3**	**4**
**Study Information**	Author(s)				
	Title				
	Publication year				
	Country				
	Methodology				
	Sample size (participants, arms, control)				
	Condition(s) included				
	Main conclusion				
**Intervention**	Description of intervention				
	Hypothesis supporting intervention design and/or proposed mechanism of action				
	Characteristics of person(s) delivering intervention (e.g., profession, clinical skills, training to deliver intervention)				
**Participant details**	Age				
	Gender				
	Ethnicity				
	Recruitment setting (e.g., primary care, secondary care etc.)				
	Other participant characteristics (e.g., time since diagnosis, patient activation level, social context, optimised pharmacological management, comorbidities)				

For peer review only

Appendix 3: Proposed search strategy

3.1 EBSCO (AMED, CINAHLPlus, MEDLINE, PsycINFO)

S1 TITLE (“musculoskeletal condition” OR “musculoskeletal disease” OR “inflammatory arthritis” OR rheumat* OR osteoarthritis OR fibromyalgia OR “juvenile idiopathic arthritis” OR “juvenile chronic arthritis” OR spondyloarth* OR “systemic sclerosis” OR “Sjogren’s syndrome” OR lupus OR “psoriatic arthritis” OR spondylitis OR scleroderma OR *myositis OR “immune-mediated necrotising myopathy” OR “anti-synthetase syndrome” OR vasculitis OR “giant cell arteritis” OR “Takayasu’s arteritis” OR “polyarteritis nodosa” OR “granulomatosis with polyangiitis” OR “microscopic angiitis” OR “Behcet syndrome” OR “Wegener syndrome” OR “Churg-Strauss syndrome” OR “low* back pain” OR “Ehlers Danlos” OR hypermobility) OR ABSTRACT (“musculoskeletal condition” OR “musculoskeletal disease” OR “inflammatory arthritis” OR rheumat* OR osteoarthritis OR fibromyalgia OR “juvenile idiopathic arthritis” OR “juvenile chronic arthritis” OR spondyloarth* OR “systemic sclerosis” OR “Sjogren’s syndrome” OR lupus OR “psoriatic arthritis” OR spondylitis OR scleroderma OR *myositis OR “immune-mediated necrotising myopathy” OR “anti-synthetase syndrome” OR vasculitis OR “giant cell arteritis” OR “Takayasu’s arteritis” OR “polyarteritis nodosa” OR “granulomatosis with polyangiitis” OR “microscopic angiitis” OR “Behcet syndrome” OR “Wegener syndrome” OR “Churg-Strauss syndrome” OR “low* back pain” OR “Ehlers Danlos” OR hypermobility)

S2 TITLE (“non-pharmacological intervention” OR “physical activity” OR exercis* OR mindfulness OR “cognitive behavioural therapy” OR “psycho-educational” OR psychosocial OR education* OR complementary OR “holistic therap*” OR nutrition OR diet OR massage OR “nurse-led” OR physiotherapy OR “occupational therapy” OR “sports rehabilitation therapy” OR “electro-physical modalities” OR thermotherapy OR “manual therapy” OR balneotherapy OR “tai chi” OR yoga OR reflexology OR aromatherapy OR chiropractic OR acupuncture OR electroacupuncture OR “low level laser therapy” OR “electric stimulation therapy” OR hyperthermia OR walk* OR pacing OR hydrotherapy OR “activity diar*” OR swim* OR “dry needl*”) OR ABSTRACT (“non-pharmacological intervention” OR “physical activity” OR exercis* OR mindfulness OR “cognitive behavioural therapy” OR “psycho-educational” OR psychosocial OR education* OR complementary OR “holistic therap*” OR nutrition OR diet OR massage OR “nurse-led” OR physiotherapy OR “occupational therapy” OR “sports rehabilitation therapy” OR “electro-physical modalities” OR thermotherapy OR “manual therapy” OR balneotherapy OR “tai chi” OR yoga OR reflexology OR aromatherapy OR chiropractic OR acupuncture OR electroacupuncture OR “low level laser therapy” OR “electric stimulation therapy” OR hyperthermia OR walk* OR pacing OR hydrotherapy OR “activity diar*” OR swim* OR “dry needl*”)

S3 TITLE (fatigue) OR ABSTRACT (fatigue)

S4 S1 AND S2 AND S3

S5 Limit S5 to;

- Publication date during or after 2007
- English language
- Peer reviewed

For peer review only

3.2 Ovid (EMBASE)

#1 (musculoskeletal condition OR musculoskeletal disease OR inflammatory arthritis OR rheumat* OR osteoarthritis OR fibromyalgia OR juvenile idiopathic arthritis OR spondyloarth* OR systemic sclerosis OR Sjogrens syndrome OR lupus OR psoriatic arthritis OR spondylitis OR scleroderma OR myositis OR immune-mediated necrotising myopathy OR anti-synthetase syndrome OR vasculitis OR giant cell arteritis OR Takayasus arteritis OR polyarteritis nodosa OR granulomatosis with polyangiitis OR microscopic angiitis OR Behcet syndrome OR Wegener syndrome OR Churg-Strauss syndrome OR low* back pain OR Ehlers Danlos OR hypermobility).ti OR (musculoskeletal condition OR musculoskeletal disease OR inflammatory arthritis OR rheumat* OR osteoarthritis OR fibromyalgia OR juvenile idiopathic arthritis OR spondyloarth* OR systemic sclerosis OR Sjogrens syndrome OR lupus OR psoriatic arthritis OR spondylitis OR scleroderma OR myositis OR immune-mediated necrotising myopathy OR anti-synthetase syndrome OR vasculitis OR giant cell arteritis OR Takayasus arteritis OR polyarteritis nodosa OR granulomatosis with polyangiitis OR microscopic angiitis OR Behcet syndrome OR Wegener syndrome OR Churg-Strauss syndrome OR low* back pain OR Ehlers Danlos OR hypermobility).ab.

#2 Limit 1 to (English language and yr=”2007”)

#3 (fatigue).ti. or (fatigue).ab.

#4 Limit 2 to (English language and yr=”2007”)

#5 (non-pharmacological intervention OR physical activity or exercis* OR mindfulness OR cognitive behavioural therapy OR psycho-educational OR psychosocial OR education* OR complementary OR holistic therp* OR nutrition OR diet OR massage OR nurse-led OR physiotherapy OR occupational therapy OR sports rehabilitation therapy OR electro-physical modalities OR thermotherapy OR manual therapy OR balneotherapy OR tai chi OR yoga OR reflexology OR aromatherapy OR chiropractic OR acupuncture OR electroacupuncture OR low level laser therapy OR electric stimulation therapy OR hyperthermia OR walk* OR pacing OR hydrotherapy OR activity diar* OR swim* OR dry needl*).ti. OR (non-pharmacological intervention OR physical activity or exercis* OR mindfulness OR cognitive behavioural therapy OR psycho-educational OR psychosocial OR education* OR complementary OR holistic therp* OR nutrition OR diet OR massage OR nurse-led OR physiotherapy OR occupational therapy OR sports rehabilitation therapy OR electro-physical modalities OR thermotherapy OR manual therapy OR balneotherapy OR tai chi OR yoga OR reflexology OR aromatherapy OR chiropractic OR acupuncture OR electroacupuncture OR low level laser therapy OR electric stimulation therapy OR hyperthermia OR walk* OR pacing OR hydrotherapy OR activity diar* OR swim* OR dry needl*).ab.

#6 Limit 5 to (English language and yr=”2007”)

#7 2 and 4 and 6

For peer review only

3.3 SCOPUS

#1 (TITLE-ABS("non pharmacological intervention" OR "physical activity" OR exercis* OR mindfulness OR "cognitive behavioural therapy" OR psycho-educational OR psychosocial OR education* OR complementary OR “holistic therap*” OR nutrition OR diet OR massage OR "nurse-led” OR physiotherapy OR "occupational therapy" OR “sports rehabilitation therapy” OR "electro-physical modalities" OR thermotherapy OR "manual therapy" OR balneotherapy OR "tai chi" OR yoga OR reflexology OR aromatherapy OR chiropractic OR acupuncture OR electroacupuncture OR "low level laser therapy" OR "electric stimulation therapy" OR hyperthermia OR walk* OR pacing OR hydrotherapy OR “activity diar*” OR swim* OR “dry needl*”)

#2 Limit #1 to:

- Publication during or after 2007
- English language

#3 TITLE-ABS(fatigue)

#4 Limit #3 to:

- Publication during or after 2007
- English language

#5 TITLE-ABS(“musculoskeletal condition” OR “musculoskeletal disease” OR “inflammatory arthritis” OR rheumat* OR osteoarthritis OR fibromyalgia OR “juvenile idiopathic arthritis” OR spondyloarth* OR “systemic sclerosis” OR “Sjogrens syndrome” OR lupus OR “psoriatic arthritis” OR spondylitis OR scleroderma OR *myositis OR “immune-mediated necrotising myopathy” OR “anti-synthetase syndrome” OR vasculitis OR “giant cell arteritis” OR “Takayasus arteritis” OR “polyarteritis nodosa” OR “granulomatosis polyangiitis” OR “microscopic angiitis” OR “Behcet syndrome” OR “Wegener syndrome” OR “Churg-Strauss syndrome” OR “low* back pain” OR “Ehlers Danlos” OR hypermobility)

#6 Limit #5 to:

- Publication during or after 2007
- English language

#7 #2 AND #4 AND #6

For peer review only

3.4 COCHRANE LIBRARY DATABASE

ID Search terms Results

#1 ("non pharmacological intervention" OR "physical activity" OR exercis* OR mindfulness OR "cognitive behavioural therapy" OR psycho-educational OR psychosocial OR education* OR complementary OR “holistic therap*” OR nutrition OR diet OR massage OR "nurse-led” OR physiotherapy OR "occupational therapy" OR “sports rehabilitation therapy” OR "electro-physical modalities" OR thermotherapy OR "manual therapy" OR balneotherapy OR "tai chi" OR yoga OR reflexology OR aromatherapy OR chiropractic OR acupuncture OR electroacupuncture OR "low level laser therapy" OR "electric stimulation therapy" OR hyperthermia OR walk* OR pacing OR hydrotherapy OR “activity diar*” OR swim* OR “dry needl*”):ti,ab

#2 (fatigue):ti,ab

#3 (“musculoskeletal condition” OR “musculoskeletal disease” OR “inflammatory arthritis” OR rheumat* OR osteoarthritis OR fibromyalgia OR “juvenile idiopathic arthritis” OR spondyloarth* OR “systemic sclerosis” OR “Sjogren’s syndrome” OR lupus OR “psoriatic arthritis” OR spondylitis OR scleroderma OR *myositis OR “immune-mediated necrotising myopathy” OR “anti-synthetase syndrome” OR vasculitis OR “giant cell arteritis” OR “Takayasu’s arteritis” OR “polyarteritis nodosa” OR “granulomatosis with polyangiitis” OR “microscopic angiitis” OR “Behcet syndrome” OR “Wegener syndrome” OR “Churg-Strauss syndrome” OR “low* back pain” OR “Ehlers Danlos” OR hypermobility):ti,ab

#4 #1 AND #2 AND #3

#5 Limit to:

- Publication date from Jan 2007 to Oct 2023
- English language

For peer review only

Preferred Reporting Items for Systematic reviews and Meta-Analyses extension for Scoping Reviews (PRISMA-ScR) Checklist

SECTION	ITEM	PRISMA-ScR CHECKLIST ITEM	REPORTED ON PAGE #
**TITLE**			
Title	1	Identify the report as a scoping review.	1
**ABSTRACT**			
Structured summary	2	Provide a structured summary that includes (as applicable): background, objectives, eligibility criteria, sources of evidence, charting methods, results, and conclusions that relate to the review questions and objectives.	1-2
**INTRODUCTION**			
Rationale	3	Describe the rationale for the review in the context of what is already known. Explain why the review questions/objectives lend themselves to a scoping review approach.	2-3
Objectives	4	Provide an explicit statement of the questions and objectives being addressed with reference to their key elements (e.g., population or participants, concepts, and context) or other relevant key elements used to conceptualize the review questions and/or objectives.	3
**METHODS**			
Protocol and registration	5	Indicate whether a review protocol exists; state if and where it can be accessed (e.g., a Web address); and if available, provide registration information, including the registration number.	n/a
Eligibility criteria	6	Specify characteristics of the sources of evidence used as eligibility criteria (e.g., years considered, language, and publication status), and provide a rationale.	4
Information sources*	7	Describe all information sources in the search (e.g., databases with dates of coverage and contact with authors to identify additional sources), as well as the date the most recent search was executed.	3
Search	8	Present the full electronic search strategy for at least 1 database, including any limits used, such that it could be repeated.	Appendix 3
Selection of sources of evidence†	9	State the process for selecting sources of evidence (i.e., screening and eligibility) included in the scoping review.	4-5
Data charting process‡	10	Describe the methods of charting data from the included sources of evidence (e.g., calibrated forms or forms that have been tested by the team before their use, and whether data charting was done independently or in duplicate) and any processes for obtaining and confirming data from investigators.	5
Data items	11	List and define all variables for which data were sought and any assumptions and simplifications made.	Appendix 2
Critical appraisal of individual sources of evidence§	12	If done, provide a rationale for conducting a critical appraisal of included sources of evidence; describe the methods used and how this information was used in any data synthesis (if appropriate).	n/a
Synthesis of results	13	Describe the methods of handling and summarizing the data that were charted.	n/a

St. Michael's
Inspired Care.
Inspiring Science.

For peer review only - http://bmjopen.bmj.com/site/about/guidelines.xhtml

SECTION	ITEM	PRISMA-ScR CHECKLIST ITEM	REPORTED ON PAGE #
**RESULTS**			
Selection of sources of evidence	14	Give numbers of sources of evidence screened, assessed for eligibility, and included in the review, with reasons for exclusions at each stage, ideally using a flow diagram.	n/a
Characteristics of sources of evidence	15	For each source of evidence, present characteristics for which data were charted and provide the citations.	n/a
Critical appraisal within sources of evidence	16	If done, present data on critical appraisal of included sources of evidence (see item 12).	n/a
Results of individual sources of evidence	17	For each included source of evidence, present the relevant data that were charted that relate to the review questions and objectives.	n/a
Synthesis of results	18	Summarize and/or present the charting results as they relate to the review questions and objectives.	n/a
**DISCUSSION**			
Summary of evidence	19	Summarize the main results (including an overview of concepts, themes, and types of evidence available), link to the review questions and objectives, and consider the relevance to key groups.	n/a
Limitations	20	Discuss the limitations of the scoping review process.	n/a
Conclusions	21	Provide a general interpretation of the results with respect to the review questions and objectives, as well as potential implications and/or next steps.	n/a
**FUNDING**			
Funding	22	Describe sources of funding for the included sources of evidence, as well as sources of funding for the scoping review. Describe the role of the funders of the scoping review.	6

JBI = Joanna Briggs Institute; PRISMA-ScR = Preferred Reporting Items for Systematic reviews and Meta-Analyses extension for Scoping Reviews.

* Where *sources of evidence* (see second footnote) are compiled from, such as bibliographic databases, social media platforms, and Web sites.

† A more inclusive/heterogeneous term used to account for the different types of evidence or data sources (e.g., quantitative and/or qualitative research, expert opinion, and policy documents) that may be eligible in a scoping review as opposed to only studies. This is not to be confused with *information sources* (see first footnote).

‡ The frameworks by Arksey and O'Malley (6) and Levac and colleagues (7) and the JBI guidance (4, 5) refer to the process of data extraction in a scoping review as data charting.

§ The process of systematically examining research evidence to assess its validity, results, and relevance before using it to inform a decision. This term is used for items 12 and 19 instead of "risk of bias" (which is more applicable to systematic reviews of interventions) to include and acknowledge the various sources of evidence that may be used in a scoping review (e.g., quantitative and/or qualitative research, expert opinion, and policy document).

From: Tricco AC, Lillie E, Zarin W, O'Brien KK, Colquhoun H, Levac D, et al. PRISMA Extension for Scoping Reviews (PRISMAScR): Checklist and Explanation. Ann Intern Med. 2018;169:467–473. doi: 10.7326/M18-0850.

St. Michael's
Inspired Care. Inspiring Science.

For peer review only - http://bmjopen.bmj.com/site/about/guidelines.xhtml

For peer review only

BMJ Open

Factors influencing the outcomes of non-pharmacological interventions for managing fatigue across the lifespan of people living with musculoskeletal (MSK) conditions: a scoping review protocol

Journal:	*BMJ Open*
Manuscript ID	bmjopen-2023-082555.R1
Article Type:	Protocol
Date Submitted by the Author:	22-Feb-2024
Complete List of Authors:	Fishpool, Katie; University of the West of England, School of Health and Social Wellbeing; University Hospitals Bristol and Weston NHS Foundation Trust, Academic Rheumatology<br>Young, George; University of the West of England<br>Ciurtin, C; University College London,<br>Cramp, Fiona; University of the West of England<br>Erhieyovwe, Emmanuel; Hywel Dda University Health Board<br>Farisogullari, Bayram; University College London<br>Macfarlane, Gary ; University of Aberdeen Aberdeen Centre for Arthritis and Musculoskeletal Health<br>Machado, Pedro M.; University College London, Centre for Rheumatology; University College London, Neuromuscular Diseases<br>Pearson, J; University of the West of England Bristol<br>Santos, Eduardo ; Hospitals of Coimbra University<br>Dures, Emma; University of the West of England; University Hospitals Bristol and Weston NHS Foundation Trust
**Primary Subject Heading**:	Rheumatology
Secondary Subject Heading:	Paediatrics, General practice / Family practice, Nursing, Evidence based practice, Complementary medicine
Keywords:	Fatigue, Musculoskeletal disorders < ORTHOPAEDIC & TRAUMA SURGERY, RHEUMATOLOGY, Paediatric rheumatology < PAEDIATRICS

SCHOLARONE™
Manuscripts

Factors influencing the outcomes of non-pharmacological interventions for managing fatigue across the lifespan of people living with musculoskeletal (MSK) conditions: a scoping review protocol

Word count: 2495

Katie Fishpool[1,2], George Young[1], Coziana Ciurtin[3], Fiona Cramp[1], Emmanuel Erhieyovwe[4], Bayram Farisogullari[5], Gary Macfarlane[6], Pedro M. Machado[5,7], Jen Pearson[1], Eduardo Santos[8], Emma Dures[1,2]

Corresponding author: Katie Fishpool katie.fishpool@uwe.ac.uk

[1] College of Health and Social Wellbeing, College of Health, Science and Society, University of the West of England, Bristol, UK

[2] Academic Rheumatology, University Hospitals Bristol and Weston NHS Foundation Trust, UK

[3] Centre for Adolescent Rheumatology, Division of Medicine, UCL Queen Square Institute of Neurology, University College London, UK

[4] General Internal Medicine, Withybush General Hospital Haverfordwest, Hywel Dda University Health Board, UK

[5] Department of Neuromuscular Diseases, University College London, UK

[6] Aberdeen Centre for Arthritis and Musculoskeletal Health (Epidemiology Group), University of Aberdeen, UK

[7] Centre for Rheumatology, Division of Medicine, University College London, UK

[8] Polytechnic University of Viseu, School of Health, Portugal; Health Sciences Research Unit: Nursing (UICISA: E); Portugal Centre for Evidence Based Practice (PCEBP): a JBI Centre of Excellence, Portugal

ABSTRACT

Introduction

Fatigue is an important and distressing symptom for many people living with chronic musculoskeletal (MSK) conditions. Many non-pharmacological interventions have been investigated in recent years and some have been demonstrated to be effective in reducing fatigue and fatigue impact, however there is limited guidance for clinicians to follow regarding the most appropriate management options. The objective of this scoping review is to understand and map the extent of evidence in relation to the factors that relate to the outcome of non-pharmacological interventions on MSK condition-related fatigue across the lifespan.

Methods and analysis

This scoping review will include evidence relating to people of all ages living with chronic MSK conditions who have been offered a non-pharmacological intervention with either the intention or effect of reducing fatigue and its impact. Databases including AMED, PsycINFO, CINAHLPlus, MEDLINE, EMBASE and Scopus will be searched for peer reviewed primary research studies published after 1st January 2007 in English language. These findings will be used to identify factors associated with successful interventions and to map gaps in knowledge.

Ethics and dissemination

For peer review only

Ethical approval was not required for this review. Findings will be disseminated by journal publication, conference presentation and by communicating with relevant healthcare and charity organisations.

Keywords: chronic musculoskeletal condition, fatigue, lifespan, non-pharmacological intervention

Article Summary

Strengths and limitations of this study

- Patient and Public Involvement and Engagement (PPIE) workshops at key time points will ensure that the protocol, review findings and subsequent discussion are relevant to stakeholders and reflect lived experience of MSK-fatigue
- All studies will be reviewed, and data extraction checked by a minimum of two researchers
- The effectiveness of specific interventions and methodological quality of included studies is not covered in this scoping review
- Only evidence available in English will be reviewed

INTRODUCTION

Musculoskeletal (MSK) conditions include inflammatory and non-inflammatory conditions such as connective tissue diseases, inflammatory arthritis and osteoarthritis, back and neck pain, and fibromyalgia which affect the muscles, bones, joints and connective tissue (1,2). More than 10 million people in the UK and 1.7 billion people globally currently live with an MSK condition (1,2). Prevalence increases with age but these conditions are encountered across the lifespan, with approximately 234,000 children in England and Scotland living with a long term MSK condition (2).

Fatigue has been identified by people living with chronic MSK conditions as a priority symptom which has a significant impact on quality of life (2–8). Pharmacological treatments are not licensed for the management of fatigue without concurrent disease activity, so the focus in clinical practice has been on non-pharmacological options (9,10). This has been mirrored in healthcare research and recent systematic reviews have examined the strength of the evidence supporting a range of non-pharmacological interventions in different patient groups (11–15). Non-pharmacological interventions are any non-chemical or biological interventions that are theoretically based and empirically proven, or that have a logical rationale which is possible to prove by empirical study (11,16).

Recent and ongoing studies on the topic of fatigue support the need for this scoping review and have been used to inform its design. A previous scoping review exploring fatigue in patients with rheumatic and MSK conditions (17) reported on the efficacy of interventions and also considered determinants associated with fatigue. A systematic review assessed the quality of evidence available to support non-pharmacological interventions to reduce fatigue in patients with inflammatory rheumatic conditions (14). The National Institute for Health and Care Research (NIHR) have recently awarded funding for the project "Effectiveness of Interventions For FatiguE in Long term conditions" (EIFFEL) (18). Study protocols have been registered on the PROSPERO database which state that the team will be conducting two systematic reviews; one to assess the effectiveness of interventions for fatigue in long term conditions (19) and the other to explore the acceptability of interventions for fatigue (20). There is potential for duplication of effort when considering acceptability of

For peer review only - http://bmjopen.bmj.com/site/about/guidelines.xhtml

interventions, as the qualitative data may explore contextual factors and some musculoskeletal conditions are also included in their searches. However, the proposed methodology of this study is a scoping review which allows for a broader view of contextual factors across the lifespan of all chronic MSK conditions and will be gathered from multiple study designs, which is appropriate for the aims of the review to map existing knowledge. Contact has been made with the EIFFEL team to reduce risk of duplication and share knowledge.

The European Alliance of Associations for Rheumatology (EULAR) recently funded a taskforce to examine the effectiveness of non-pharmacological interventions for fatigue in inflammatory rheumatic conditions and the resulting systematic review found strong evidence that some interventions are effective (14). This informed the EULAR recommendations for the management of fatigue (21) and highlighted a need to better understand contextual factors and the mechanisms by which interventions are effective (7). There is currently no comprehensive understanding of the factors which influence the success of an intervention. The impact of this is a lack of evidence to support decision making in how to design, offer and deliver interventions in the most effective way, tailored to a range of patients and at the optimal time. Current clinical guidelines for the management of common MSK conditions recognise fatigue as an important symptom but do not make any recommendations for how it can be addressed directly (22–24), hindering implementation of the evidence.

The clinical pathway for management of MSK conditions in the UK differs depending on primary diagnosis, with suspected inflammatory conditions being referred to specialist secondary care settings and osteoarthritis and fibromyalgia being managed predominantly through primary care (24–26). The impact of this is that the experience of patients with MSK fatigue and the profession and skills of the clinicians who provide their care may be quite different.

Review aims

The aim of this scoping review is to generate evidence for health professionals and educators to design or adapt tailored MSK-fatigue support. The objectives of this review are to identify evidence for existing interventions for MSK-fatigue across the life course and to explore the theoretical basis for the interventions. To explore the comprehensive nature of the existing evidence, the clinical and demographic characteristics of the participants as well as to capture the training/skills of those who deliver the interventions where this information is available. As the intention is to create an overview of the current knowledge and to highlight gaps in the existing literature rather than to assess the effectiveness of specific interventions, a scoping review is the most appropriate methodological approach (27,28).

METHODS AND ANALYSIS

Study design

In accordance with the Joanna Briggs Institute (JBI) methodology for scoping reviews (29), this protocol sets out the criteria that the reviewing team will use to include and exclude sources of evidence and to identify what data is relevant. The data will be reported using the PRISMA-ScR extension guidelines for scoping reviews (30).

Identifying relevant studies

For peer review only

A preliminary search of MEDLINE in August 2023 identified the current scope and scale of the evidence base related to the scoping review. The search strategy (Appendix 1) was then developed with support from a specialist subject librarian and reviewed by stakeholders in a PPIE workshop.

The following electronic databases will be searched for research published in peer-reviewed journals from January 2007 onwards; AMED, MEDLINE, PsycINFO, CINAHLPlus (EBSCO platform), EMBASE (Ovid platform), SCOPUS and Cochrane Database. This date was chosen to correspond with the Outcome Measures in Rheumatology 8th meeting (OMERACT 8) which endorsed fatigue as an addition to the 'core set' of outcome measures for all subsequent studies, highlighting the importance of investigating this symptom (31). There will be no restrictions on the age of participants, allowing interventions that have been used throughout the life course and highlighting any gaps in provision. A search for unpublished studies will not be conducted due to limitations of time to complete this review. The reference lists from included studies will be hand searched to check for any other relevant papers not captured in the database searching. Only evidence available in English will be reviewed and studies in other languages will be excluded due to time. All studies will be uploaded to the review software system Covidence (32).

Selection of studies

A minimum of two independent reviewers will screen the titles and abstracts of all identified studies against the stated inclusion and exclusion criteria (Table 1). Regular meetings will be held by the team throughout the title and abstract screening process to aid understanding and reduce disagreements (33). Papers that proceed to the full text stage of screening will also be reviewed by two or more independent reviewers who will document the main reason any excluded papers do not meet the inclusion criteria. Any differences in opinion between the reviewers will be resolved by discussing the papers, with an additional independent reviewer to support mediation, as required.

Table 1: Inclusion and exclusion criteria

Inclusion criteria	Exclusion criteria
- Primary research study<br>- Published in a peer reviewed journal<br>- Available in English language<br>- Participants have one or more chronic MSK conditions<br>- Participants experience fatigue at baseline<br>- Published during or after 2007<br>- Describes a non-pharmacological intervention to manage MSK condition symptoms, with fatigue reduction as a primary or secondary outcome	- Reviews, protocols, opinion pieces, editorials, case series, case reports, observational cohort studies<br>- Pharmacological interventions<br>- No intervention is described<br>- Muscle fatigue rather than global fatigue is examined<br>- No data is available on factors associated with intervention success (theoretical mechanism of intervention OR characteristics of participants OR characteristics of clinicians delivering interventions)

All primary research methodologies will be considered, including experimental and quasi-experimental study designs, before and after studies and interrupted timeseries studies, analytical observational studies, case-control studies and analytical cross-sectional studies, and descriptive observational study designs. Qualitative studies will also be considered including, for example, phenomenology, grounded theory, ethnography, qualitative description, and action research.

Data extraction

For peer review only - http://bmjopen.bmj.com/site/about/guidelines.xhtml

Data from included papers will be extracted by one reviewer and checked by another using an adapted version of the JBI template for data extraction (Appendix 2). This template captures information about study participants, methods, and findings relevant to the research question (34) and has been amended to extract additional data on contextual factors including clinical characteristics, information on clinicians delivering interventions and the hypothesis behind the design of the intervention. Any disagreements that may arise between the reviewers will be resolved by consensus, with an additional reviewer to support mediation. If necessary, the authors of the included papers will be contacted for further information or data clarification.

Data reporting and analysis

Findings will be presented in a PRISMA (Preferred Reporting Items for Systematic Reviews and Meta Analyses) flow diagram (35) to demonstrate the number of articles identified and their sources, with reasons for exclusion at full text screening summarised. All included studies will be summarised in tabular format. Further figures will be used to illustrate a map of the existing literature and any gaps highlighted by the review. Data analysis is likely to be narrative due to the broad range of study types being included. This may be further refined for use during the review process.

Patient and public involvement statement

A patient and public involvement and engagement (PPIE) workshop was held in October 2023 during the design stage of the search strategy and review protocol. Stakeholders attending the workshop included patients living with one or more MSK conditions and clinicians from a range of professions who support patients that experience MSK related fatigue. The discussion focussed on people's experiences of offering or being offered support to manage their fatigue and asking for comments on the proposed review. This highlighted additional intervention types and pathways that were subsequently included in the search terms (Appendix 3). It also confirmed our understanding that fatigue is a significant issue and that an overview of potential interventions and management techniques would be welcomed by patients and clinicians.

Further PPIE events are planned at key points during the project to ensure the validity of the final review. Workshops that focus on the support of adults and of children and young people will be held to discuss the initial findings following data extraction with the aim of highlighting key themes and gaps in knowledge. A further pair of workshops will be arranged following synthesis of the findings to ensure the validity of the review, discuss priorities for future research and to promote dissemination of the findings through appropriate groups. The outcome of these events and how they influence the scoping review process will be reported in the final review following the guidelines for the GRIPP2 short form reporting checklist (36), which is a tool designed to improve the reporting of public and patient involvement in research.

ETHICS AND DISSEMINATION

Ethical approval is not required for this scoping review. The findings of this review will be disseminated via relevant peer-reviewed journals, conference presentations and through sharing findings with relevant charities and health professionals.

AUTHOR CONTRIBUTIONS

For peer review only

Project funding application by ED with support from FC, JP, GM, PM, CC, BF and ES. Drafting of protocol and search strategy by KF in discussion with ED and GY, reviewed by EE, FC, JP, GM, PM, CC, BF and ES.

FUNDING STATEMENT

This scoping review protocol is part of the project "MusculoskelEtal faTigue acRoss the lIfe CourSe: understanding what helps and mapping what is missing (METRICS)" (reference 23140), which is jointly funded by Versus Arthritis and The Kennedy Trust.

COMPETING INTERESTS STATEMENT

PMM has received consulting/speaker's fees from Abbvie, BMS, Celgene, Eli Lilly, Janssen, MSD, Novartis, Orphazyme, Pfizer, Roche and UCB, all unrelated to this project. There are no competing interests in this project.

ACKNOWLEDGEMENTS

The development of this scoping review search strategy was supported by Specialist Subject Librarian Pauline Shaw from the University of the West of England library. The review team is also grateful for the contributions of the stakeholders who attended the Patient and Public Involvement and Engagement workshop.

For peer review only

References

1. World Health Organisation (WHO). World Health Organisation News Room Fact Sheets. 2022 [cited 2023 Oct 17]. Musculoskeletal health. Available from: https://www.who.int/news-room/fact-sheets/detail/musculoskeletal-conditions

2. Versus Arthritis [Internet]. [cited 2023 Oct 17]. The State of Musculoskeletal Health. Available from: https://www.versusarthritis.org/about-arthritis/data-and-statistics/the-state-of-musculoskeletal-health/

3. L M M Steunebrink, M A H Oude Voshaar, E Taal, H E Vonkeman, T R Zijlstra, M A F J Van de Laar. Determinants of Perceived Health Nonimprovement in Early Rheumatoid Arthritis Patients With Favorable Treatment Outcomes. Arthritis Care Res. 2018 Apr;70(4):510–5.

4. Van Tuyl LHD, Sadlonova M, Hewlett S, Davis B, Flurey C, Goel N, et al. The patient perspective on absence of disease activity in rheumatoid arthritis: a survey to identify key domains of patient-perceived remission. Ann Rheum Dis. 2017 May;76(5):855–61.

5. Dean LE, Macfarlane GJ, Jones GT. Five Potentially Modifiable Factors Predict Poor Quality of Life in Ankylosing Spondylitis: Results from the Scotland Registry for Ankylosing Spondylitis. J Rheumatol. 2018 Jan;45(1):62–9.

6. Primdahl J, Hegelund A, Lorenzen AG, Loeppenthin K, Dures E, Appel Esbensen B. The Experience of people with rheumatoid arthritis living with fatigue: a qualitative metasynthesis. BMJ Open. 2019 Mar;9(3):e024338.

7. Kant-Smits K, Van Brussel M, Nijhof S, Van der Net J. Reducing fatigue in pediatric rheumatic conditions: a systematic review. Pediatr Rheumatol. 2021 Jul 8;19(1):111.

8. Tollisen A, Selvaag AM, Aasland A, Ingebrigtsen T, Sagen J, Lerdal A, et al. Personally Generated Quality of Life Outcomes in Adults With Juvenile Idiopathic Arthritis. J Rheumatol. 2022 Oct 1;49(10):1138–45.

9. Druce KL, Jones GT, Macfarlane GJ, Basu N. Determining Pathways to Improvements in Fatigue in Rheumatoid Arthritis: Results From the British Society for Rheumatology Biologics Register for Rheumatoid Arthritis. Arthritis Rheumatol. 2015;67(9):2303–10.

10. Salmon VE, Hewlett S, Walsh NE, Kirwan JR, Morris M, Urban M, et al. Developing a group intervention to manage fatigue in rheumatoid arthritis through modifying physical activity. BMC Musculoskelet Disord. 2019 May 4;20(1):194.

11. Cramp F, Hewlett S, Almeida C, Kirwan JR, Choy EH, Chalder T, et al. Non-pharmacological interventions for fatigue in rheumatoid arthritis. Cochrane Database Syst Rev [Internet]. 2013 [cited 2023 Sep 5];(8). Available from: https://www.readcube.com/articles/10.1002%2F14651858.cd008322.pub2

12. Bidonde J, Busch AJ, Schachter CL, Webber SC, Musselman KE, Overend TJ, et al. Mixed exercise training for adults with fibromyalgia. Cochrane Database Syst Rev [Internet]. 2019 [cited 2023 Sep 11];(5). Available from: https://www.cochranelibrary.com/cdsr/doi/10.1002/14651858.CD013340/full?highlightAbstract=fatigu%7Cfatigue

For peer review only

13. Regnaux JP, Davergne T, Palazzo C, Roren A, Rannou F, Boutron I, et al. Exercise programmes for ankylosing spondylitis. Cochrane Database Syst Rev [Internet]. 2019 [cited 2023 Sep 11];(10). Available from: https://www.cochranelibrary.com/cdsr/doi/10.1002/14651858.CD011321.pub2/full

14. Santos EJF, Farisogullari B, Dures E, Geenen R, Machado PM, EULAR taskforce on recommendations for the management of fatigue in people with inflammatory rheumatic diseases. Efficacy of non-pharmacological interventions: a systematic review informing the 2023 EULAR recommendations for the management of fatigue in people with inflammatory rheumatic and musculoskeletal diseases. RMD Open. 2023 Aug;9(3):e003350.

15. Frade S, O'Neill S, Greene D, Nutter E, Cameron M. Exercise as adjunctive therapy for systemic lupus erythematosus. Cochrane Database Syst Rev [Internet]. 2023 [cited 2023 Sep 11];(4). Available from: https://www.cochranelibrary.com/cdsr/doi/10.1002/14651858.CD014816.pub2/full?highlightAbstract=fatigu%7Cfatigue

16. Olazarán J, Reisberg B, Clare L, Cruz I, Peña-Casanova J, Del Ser T, et al. Nonpharmacological Therapies in Alzheimer's Disease: A Systematic Review of Efficacy. Dement Geriatr Cogn Disord. 2010;30(2):161–78.

17. Beckers E, Hermans K, Tubergen AV, Boonen A. Fatigue in patients with rheumatic and musculoskeletal diseases: a scoping review on definitions, measurement instruments, determinants, consequences and interventions. RMD Open. 2023 Aug 1;9(3):e003056.

18. Effectiveness of Interventions For Fatigue in Long term conditions (EIFFEL) - NIHR Funding and Awards [Internet]. [cited 2023 Oct 10]. Available from: https://fundingawards.nihr.ac.uk/award/NIHR154660

19. Joanna Leaviss, Christopher Burton, Andrew Booth, Shije Ren, Anthea Sutton, Kate Fryer, et al. (Protocol0 Effectivenesss of Interventions For Fatigue in Long term conditions (EIFFEL) [Internet]. 2023 [cited 2023 Oct 11]. Available from: https://www.crd.york.ac.uk/PROSPERO/display_record.php?RecordID=440141

20. Joanna Leaviss, Andrew Booth, Chris Burton, Sutton A, Kate Fryer, Helen Dawes, et al. (Protocol) Acceptability of Interventions For Fatigue in Long term conditions (EIFFEL) [Internet]. 2023 [cited 2023 Oct 16]. Available from: https://www.crd.york.ac.uk/PROSPERO/display_record.php?RecordID=459361

21. Dures E, Farisogullari B, Santos E, Moltó A, Feldthusen C, Harris C, et al. Pos0370 2023 Eular Recommendations for the Management of Fatigue in People with Inflammatory Rheumatic and Musculoskeletal Diseases. Ann Rheum Dis. 2023 Jun 1;82(Suppl 1):437–8.

22. Recommendations | Rheumatoid arthritis in adults: management | Guidance | NICE [Internet]. NICE; 2018 [cited 2023 Oct 11]. Available from: https://www.nice.org.uk/guidance/ng100/chapter/Recommendations

23. Recommendations | Spondyloarthritis in over 16s: diagnosis and management | Guidance | NICE [Internet]. NICE; 2017 [cited 2023 Oct 11]. Available from: https://www.nice.org.uk/guidance/NG65/chapter/Recommendations#recognition-and-referral-in-non-specialist-care-settings

24. Recommendations | Chronic pain (primary and secondary) in over 16s: assessment of all chronic pain and management of chronic primary pain | Guidance | NICE [Internet]. NICE; 2021

For peer review only

[cited 2023 Oct 10]. Available from: https://www.nice.org.uk/guidance/ng193/chapter/Recommendations#managing-chronic-primary-pain

25. NICE [Internet]. NICE; [cited 2023 Oct 10]. Management of inflammatory arthritis | NICE impact arthritis | Reviewing the impact of our guidance | Measuring the use of NICE guidance | Into practice | What we do | About. Available from: https://www.nice.org.uk/about/what-we-do/into-practice/measuring-the-use-of-nice-guidance/impact-of-our-guidance/nice-impact-arthritis/management-of-inflammatory-arthritis

26. RCP London [Internet]. 2022 [cited 2023 Oct 10]. The diagnosis of fibromyalgia syndrome. Available from: https://www.rcplondon.ac.uk/guidelines-policy/diagnosis-fibromyalgia-syndrome

27. Peters MDJ, Marnie C, Tricco AC, Pollock D, Munn Z, Alexander L, et al. Updated methodological guidance for the conduct of scoping reviews. JBI Evid Implement. 2021 Mar;19(1):3–10.

28. Munn Z, Peters MDJ, Stern C, Tufanaru C, McArthur A, Aromataris E. Systematic review or scoping review? Guidance for authors when choosing between a systematic or scoping review approach. BMC Med Res Methodol. 2018 Nov 19;18(1):143.

29. Peters MDJ, Godfrey C, McInerney P, Tricco AC, Khalil H. Chapter 11: Scoping reviews. In: JBI Manual for Evidence Synthesis [Internet]. JBI; 2020 [cited 2023 Sep 5]. Available from: https://synthesismanual.jbi.global

30. Tricco AC, Lillie E, Zarin W, O'Brien KK, Colquhoun H, Levac D, et al. PRISMA Extension for Scoping Reviews (PRISMA-ScR): Checklist and Explanation. Ann Intern Med. 2018 Oct 2;169(7):467–73.

31. Kirwan JR, Minnock P, Adebajo A, Bresnihan B, Choy E, Wit M de, et al. Patient perspective: fatigue as a recommended patient centered outcome measure in rheumatoid arthritis. J Rheumatol. 2007 May 1;34(5):1174–7.

32. Covidence [Internet]. Melbourne, Australia: Veritas Health Innovation; (Covidence systematic review software). Available from: www.covidence.org

33. Polanin JR, Pigott TD, Espelage DL, Grotpeter JK. Best practice guidelines for abstract screening large-evidence systematic reviews and meta-analyses. Res Synth Methods. 2019;10(3):330–42.

34. Appendix 11.1 JBI template source of evidence details, characteristics and results extraction instrument - JBI Manual for Evidence Synthesis - JBI Global Wiki [Internet]. [cited 2023 Oct 16]. Available from: https://jbi-global-wiki.refined.site/space/MANUAL/4687579/Appendix+11.1+JBI+template+source+of+evidence+details%2C+characteristics+and+results+extraction+instrument

35. PRISMA [Internet]. [cited 2023 Oct 16]. Available from: http://prisma-statement.org/PRISMAStatement/FlowDiagram

36. Staniszewska S, Brett J, Simera I, Seers K, Mockford C, Goodlad S, et al. GRIPP2 reporting checklists: tools to improve reporting of patient and public involvement in research. BMJ. 2017 Aug 2;358:j3453.

For peer review only

Appendix 1: Search strategy overview

	Population	Exposure	Outcome
**Search terms**	musculoskeletal condition<br>musculoskeletal disease<br>inflammatory arthritis<br>rheumat*<br>osteoarthritis<br>fibromyalgia<br>juvenile idiopathic arthritis<br>juvenile chronic arthritis<br>spondyloarth*<br>systemic sclerosis<br>Sjogren's syndrome<br>lupus<br>psoriatic arthritis<br>spondylitis<br>scleroderma<br>*myositis<br>immune mediated necrotising myopathy<br>anti synthetase syndrome<br>vasculitis<br>giant cell arteritis<br>Takayasu's arteritis<br>polyarteritis nodosa<br>granulomatosis with polyangiitis<br>microscopic angiitis<br>Behcet syndrome<br>Wegener syndrome<br>Churg-Strauss syndrome<br>low* back pain<br>Ehlers Danlos<br>hypermobility	non-pharmacological intervention<br>physical activity<br>exercis*<br>mindfulness<br>cognitive behavioural therapy<br>psycho-educational<br>psychosocial<br>education*<br>complementary<br>holistic therap*<br>nutrition<br>diet<br>massage<br>nurse-led<br>physiotherapy<br>occupational therapy<br>electro-physical modalities<br>thermotherapy<br>manual therapy<br>balneotherapy<br>tai chi<br>yoga<br>reflexology<br>aromatherapy<br>chiropractic<br>acupuncture<br>electroacupuncture<br>low level laser therapy<br>electric stimulation therapy<br>hyperthermia<br>walk*<br>pacing<br>hydrotherapy<br>activity diar*<br>swim*<br>dry needl*	fatigue
**Databases**	- EBSCO (AMED, CINAHLPlus, MEDLINE, PsycINFO)<br>- Ovid (Embase)<br>- Scopus<br>- Cochrane Database		
**Inclusion criteria**	- Primary research study<br>- Published in a peer reviewed journal<br>- Available in English language<br>- Participants have one or more chronic musculoskeletal conditions<br>- Participants experience fatigue at baseline<br>- Published during or after 2007<br>- Describes an intervention to manage MSK condition symptoms, with fatigue reduction as a primary or secondary outcome		

For peer review only

**Exclusion criteria**	- Reviews, protocols, opinion pieces, editorials, case reports, case series, observational cohort studies<br>- Pharmacological interventions<br>- No intervention is described<br>- Muscle fatigue rather than global fatigue is examined<br>- No data is available on factors associated with intervention success (theoretical mechanism of intervention OR characteristics of participants OR characteristics of clinicians delivering interventions)

For peer review only

Appendix 2: Amended JBI data extraction table

	**Study number**	**1**	**2**	**3**	**4**
**Study Information**	Author(s)				
	Title				
	Publication year				
	Country				
	Methodology				
	Sample size (participants, arms, control)				
	Condition(s) included				
	Main conclusion				
**Intervention**	Description of intervention				
	Hypothesis supporting intervention design and/or proposed mechanism of action				
	Characteristics of person(s) delivering intervention (e.g., profession, clinical skills, training to deliver intervention)				
**Participant details**	Age				
	Gender				
	Ethnicity				
	Recruitment setting (e.g., primary care, secondary care etc.)				
	Other participant characteristics (e.g., time since diagnosis, patient activation level, social context, optimised pharmacological management, comorbidities)				

For peer review only

Appendix 3: Proposed search strategy

3.1 EBSCO (AMED, CINAHLPlus, MEDLINE, PsycINFO)

S1 TITLE (“musculoskeletal condition” OR “musculoskeletal disease” OR “inflammatory arthritis” OR rheumat* OR osteoarthritis OR fibromyalgia OR “juvenile idiopathic arthritis” OR “juvenile chronic arthritis” OR spondyloarth* OR “systemic sclerosis” OR “Sjogren’s syndrome” OR lupus OR “psoriatic arthritis” OR spondylitis OR scleroderma OR *myositis OR “immune-mediated necrotising myopathy” OR “anti-synthetase syndrome” OR vasculitis OR “giant cell arteritis” OR “Takayasu’s arteritis” OR “polyarteritis nodosa” OR “granulomatosis with polyangiitis” OR “microscopic angiitis” OR “Behcet syndrome” OR “Wegener syndrome” OR “Churg-Strauss syndrome” OR “low* back pain” OR “Ehlers Danlos” OR hypermobility) OR ABSTRACT (“musculoskeletal condition” OR “musculoskeletal disease” OR “inflammatory arthritis” OR rheumat* OR osteoarthritis OR fibromyalgia OR “juvenile idiopathic arthritis” OR “juvenile chronic arthritis” OR spondyloarth* OR “systemic sclerosis” OR “Sjogren’s syndrome” OR lupus OR “psoriatic arthritis” OR spondylitis OR scleroderma OR *myositis OR “immune-mediated necrotising myopathy” OR “anti-synthetase syndrome” OR vasculitis OR “giant cell arteritis” OR “Takayasu’s arteritis” OR “polyarteritis nodosa” OR “granulomatosis with polyangiitis” OR “microscopic angiitis” OR “Behcet syndrome” OR “Wegener syndrome” OR “Churg-Strauss syndrome” OR “low* back pain” OR “Ehlers Danlos” OR hypermobility)

S2 TITLE (“non-pharmacological intervention” OR “physical activity” OR exercis* OR mindfulness OR “cognitive behavioural therapy” OR “psycho-educational” OR psychosocial OR education* OR complementary OR “holistic therap*” OR nutrition OR diet OR massage OR “nurse-led” OR physiotherapy OR “occupational therapy” OR “sports rehabilitation therapy” OR “electro-physical modalities” OR thermotherapy OR “manual therapy” OR balneotherapy OR “tai chi” OR yoga OR reflexology OR aromatherapy OR chiropractic OR acupuncture OR electroacupuncture OR “low level laser therapy” OR “electric stimulation therapy” OR hyperthermia OR walk* OR pacing OR hydrotherapy OR “activity diar*” OR swim* OR “dry needl*”) OR ABSTRACT (“non-pharmacological intervention” OR “physical activity” OR exercis* OR mindfulness OR “cognitive behavioural therapy” OR “psycho-educational” OR psychosocial OR education* OR complementary OR “holistic therap*” OR nutrition OR diet OR massage OR “nurse-led” OR physiotherapy OR “occupational therapy” OR “sports rehabilitation therapy” OR “electro-physical modalities” OR thermotherapy OR “manual therapy” OR balneotherapy OR “tai chi” OR yoga OR reflexology OR aromatherapy OR chiropractic OR acupuncture OR electroacupuncture OR “low level laser therapy” OR “electric stimulation therapy” OR hyperthermia OR walk* OR pacing OR hydrotherapy OR “activity diar*” OR swim* OR “dry needl*”)

S3 TITLE (fatigue) OR ABSTRACT (fatigue)

S4 S1 AND S2 AND S3

S5 Limit S5 to;

- Publication date during or after 2007
- English language
- Peer reviewed

For peer review only

3.2 Ovid (EMBASE)

#1 (musculoskeletal condition OR musculoskeletal disease OR inflammatory arthritis OR rheumat* OR osteoarthritis OR fibromyalgia OR juvenile idiopathic arthritis OR spondyloarth* OR systemic sclerosis OR Sjogrens syndrome OR lupus OR psoriatic arthritis OR spondylitis OR scleroderma OR myositis OR immune-mediated necrotising myopathy OR anti-synthetase syndrome OR vasculitis OR giant cell arteritis OR Takayasus arteritis OR polyarteritis nodosa OR granulomatosis with polyangiitis OR microscopic angiitis OR Behcet syndrome OR Wegener syndrome OR Churg-Strauss syndrome OR low* back pain OR Ehlers Danlos OR hypermobility).ti OR (musculoskeletal condition OR musculoskeletal disease OR inflammatory arthritis OR rheumat* OR osteoarthritis OR fibromyalgia OR juvenile idiopathic arthritis OR spondyloarth* OR systemic sclerosis OR Sjogrens syndrome OR lupus OR psoriatic arthritis OR spondylitis OR scleroderma OR myositis OR immune-mediated necrotising myopathy OR anti-synthetase syndrome OR vasculitis OR giant cell arteritis OR Takayasus arteritis OR polyarteritis nodosa OR granulomatosis with polyangiitis OR microscopic angiitis OR Behcet syndrome OR Wegener syndrome OR Churg-Strauss syndrome OR low* back pain OR Ehlers Danlos OR hypermobility).ab.

#2 Limit 1 to (English language and yr="2007")

#3 (fatigue).ti. or (fatigue).ab.

#4 Limit 2 to (English language and yr="2007")

#5 (non-pharmacological intervention OR physical activity or exercis* OR mindfulness OR cognitive behavioural therapy OR psycho-educational OR psychosocial OR education* OR complementary OR holistic therp* OR nutrition OR diet OR massage OR nurse-led OR physiotherapy OR occupational therapy OR sports rehabilitation therapy OR electro-physical modalities OR thermotherapy OR manual therapy OR balneotherapy OR tai chi OR yoga OR reflexology OR aromatherapy OR chiropractic OR acupuncture OR electroacupuncture OR low level laser therapy OR electric stimulation therapy OR hyperthermia OR walk* OR pacing OR hydrotherapy OR activity diar* OR swim* OR dry needl*).ti. OR (non-pharmacological intervention OR physical activity or exercis* OR mindfulness OR cognitive behavioural therapy OR psycho-educational OR psychosocial OR education* OR complementary OR holistic therp* OR nutrition OR diet OR massage OR nurse-led OR physiotherapy OR occupational therapy OR sports rehabilitation therapy OR electro-physical modalities OR thermotherapy OR manual therapy OR balneotherapy OR tai chi OR yoga OR reflexology OR aromatherapy OR chiropractic OR acupuncture OR electroacupuncture OR low level laser therapy OR electric stimulation therapy OR hyperthermia OR walk* OR pacing OR hydrotherapy OR activity diar* OR swim* OR dry needl*).ab.

#6 Limit 5 to (English language and yr="2007")

#7 2 and 4 and 6

For peer review only

3.3 SCOPUS

#1 (TITLE-ABS("non pharmacological intervention" OR "physical activity" OR exercis* OR mindfulness OR "cognitive behavioural therapy" OR psycho-educational OR psychosocial OR education* OR complementary OR “holistic therap*” OR nutrition OR diet OR massage OR "nurse-led” OR physiotherapy OR "occupational therapy" OR “sports rehabilitation therapy” OR "electro-physical modalities" OR thermotherapy OR "manual therapy" OR balneotherapy OR "tai chi" OR yoga OR reflexology OR aromatherapy OR chiropractic OR acupuncture OR electroacupuncture OR "low level laser therapy" OR "electric stimulation therapy" OR hyperthermia OR walk* OR pacing OR hydrotherapy OR “activity diar*” OR swim* OR “dry needl*”)

#2 Limit #1 to:

- Publication during or after 2007
- English language

#3 TITLE-ABS(fatigue)

#4 Limit #3 to:

- Publication during or after 2007
- English language

#5 TITLE-ABS(“musculoskeletal condition” OR “musculoskeletal disease” OR “inflammatory arthritis” OR rheumat* OR osteoarthritis OR fibromyalgia OR “juvenile idiopathic arthritis” OR spondyloarth* OR “systemic sclerosis” OR “Sjogrens syndrome” OR lupus OR “psoriatic arthritis” OR spondylitis OR scleroderma OR *myositis OR “immune-mediated necrotising myopathy” OR “anti-synthetase syndrome” OR vasculitis OR “giant cell arteritis” OR “Takayasus arteritis” OR “polyarteritis nodosa” OR “granulomatosis polyangiitis” OR “microscopic angiitis” OR “Behcet syndrome” OR “Wegener syndrome” OR “Churg-Strauss syndrome” OR “low* back pain” OR “Ehlers Danlos” OR hypermobility)

#6 Limit #5 to:

- Publication during or after 2007
- English language

#7 #2 AND #4 AND #6

For peer review only

3.4 COCHRANE LIBRARY DATABASE

ID Search terms Results

#1 ("non pharmacological intervention" OR "physical activity" OR exercis* OR mindfulness OR "cognitive behavioural therapy" OR psycho-educational OR psychosocial OR education* OR complementary OR “holistic therap*” OR nutrition OR diet OR massage OR "nurse-led” OR physiotherapy OR "occupational therapy" OR “sports rehabilitation therapy” OR "electro-physical modalities" OR thermotherapy OR "manual therapy" OR balneotherapy OR "tai chi" OR yoga OR reflexology OR aromatherapy OR chiropractic OR acupuncture OR electroacupuncture OR "low level laser therapy" OR "electric stimulation therapy" OR hyperthermia OR walk* OR pacing OR hydrotherapy OR “activity diar*” OR swim* OR “dry needl*”):ti,ab

#2 (fatigue):ti,ab

#3 (“musculoskeletal condition” OR “musculoskeletal disease” OR “inflammatory arthritis” OR rheumat* OR osteoarthritis OR fibromyalgia OR “juvenile idiopathic arthritis” OR spondyloarth* OR “systemic sclerosis” OR “Sjogren’s syndrome” OR lupus OR “psoriatic arthritis” OR spondylitis OR scleroderma OR *myositis OR “immune-mediated necrotising myopathy” OR “anti-synthetase syndrome” OR vasculitis OR “giant cell arteritis” OR “Takayasu’s arteritis” OR “polyarteritis nodosa” OR “granulomatosis with polyangiitis” OR “microscopic angiitis” OR “Behcet syndrome” OR “Wegener syndrome” OR “Churg-Strauss syndrome” OR “low* back pain” OR “Ehlers Danlos” OR hypermobility):ti,ab

#4 #1 AND #2 AND #3

#5 Limit to:

- Publication date from Jan 2007 to Oct 2023
- English language

For peer review only

Preferred Reporting Items for Systematic reviews and Meta-Analyses extension for Scoping Reviews (PRISMA-ScR) Checklist

SECTION	ITEM	PRISMA-ScR CHECKLIST ITEM	REPORTED ON PAGE #
**TITLE**			
Title	1	Identify the report as a scoping review.	1
**ABSTRACT**			
Structured summary	2	Provide a structured summary that includes (as applicable): background, objectives, eligibility criteria, sources of evidence, charting methods, results, and conclusions that relate to the review questions and objectives.	1-2
**INTRODUCTION**			
Rationale	3	Describe the rationale for the review in the context of what is already known. Explain why the review questions/objectives lend themselves to a scoping review approach.	2-3
Objectives	4	Provide an explicit statement of the questions and objectives being addressed with reference to their key elements (e.g., population or participants, concepts, and context) or other relevant key elements used to conceptualize the review questions and/or objectives.	3
**METHODS**			
Protocol and registration	5	Indicate whether a review protocol exists; state if and where it can be accessed (e.g., a Web address); and if available, provide registration information, including the registration number.	n/a
Eligibility criteria	6	Specify characteristics of the sources of evidence used as eligibility criteria (e.g., years considered, language, and publication status), and provide a rationale.	4
Information sources*	7	Describe all information sources in the search (e.g., databases with dates of coverage and contact with authors to identify additional sources), as well as the date the most recent search was executed.	3
Search	8	Present the full electronic search strategy for at least 1 database, including any limits used, such that it could be repeated.	Appendix 3
Selection of sources of evidence†	9	State the process for selecting sources of evidence (i.e., screening and eligibility) included in the scoping review.	4-5
Data charting process‡	10	Describe the methods of charting data from the included sources of evidence (e.g., calibrated forms or forms that have been tested by the team before their use, and whether data charting was done independently or in duplicate) and any processes for obtaining and confirming data from investigators.	5
Data items	11	List and define all variables for which data were sought and any assumptions and simplifications made.	Appendix 2
Critical appraisal of individual sources of evidence§	12	If done, provide a rationale for conducting a critical appraisal of included sources of evidence; describe the methods used and how this information was used in any data synthesis (if appropriate).	n/a
Synthesis of results	13	Describe the methods of handling and summarizing the data that were charted.	n/a

For peer review only - http://bmjopen.bmj.com/site/about/guidelines.xhtml

SECTION	ITEM	PRISMA-ScR CHECKLIST ITEM	REPORTED ON PAGE #
**RESULTS**			
Selection of sources of evidence	14	Give numbers of sources of evidence screened, assessed for eligibility, and included in the review, with reasons for exclusions at each stage, ideally using a flow diagram.	n/a
Characteristics of sources of evidence	15	For each source of evidence, present characteristics for which data were charted and provide the citations.	n/a
Critical appraisal within sources of evidence	16	If done, present data on critical appraisal of included sources of evidence (see item 12).	n/a
Results of individual sources of evidence	17	For each included source of evidence, present the relevant data that were charted that relate to the review questions and objectives.	n/a
Synthesis of results	18	Summarize and/or present the charting results as they relate to the review questions and objectives.	n/a
**DISCUSSION**			
Summary of evidence	19	Summarize the main results (including an overview of concepts, themes, and types of evidence available), link to the review questions and objectives, and consider the relevance to key groups.	n/a
Limitations	20	Discuss the limitations of the scoping review process.	n/a
Conclusions	21	Provide a general interpretation of the results with respect to the review questions and objectives, as well as potential implications and/or next steps.	n/a
**FUNDING**			
Funding	22	Describe sources of funding for the included sources of evidence, as well as sources of funding for the scoping review. Describe the role of the funders of the scoping review.	6

JBI = Joanna Briggs Institute; PRISMA-ScR = Preferred Reporting Items for Systematic reviews and Meta-Analyses extension for Scoping Reviews.

* Where *sources of evidence* (see second footnote) are compiled from, such as bibliographic databases, social media platforms, and Web sites.

† A more inclusive/heterogeneous term used to account for the different types of evidence or data sources (e.g., quantitative and/or qualitative research, expert opinion, and policy documents) that may be eligible in a scoping review as opposed to only studies. This is not to be confused with *information sources* (see first footnote).

‡ The frameworks by Arksey and O'Malley (6) and Levac and colleagues (7) and the JBI guidance (4, 5) refer to the process of data extraction in a scoping review as data charting.

§ The process of systematically examining research evidence to assess its validity, results, and relevance before using it to inform a decision. This term is used for items 12 and 19 instead of "risk of bias" (which is more applicable to systematic reviews of interventions) to include and acknowledge the various sources of evidence that may be used in a scoping review (e.g., quantitative and/or qualitative research, expert opinion, and policy document).

From: Tricco AC, Lillie E, Zarin W, O'Brien KK, Colquhoun H, Levac D, et al. PRISMA Extension for Scoping Reviews (PRISMAScR): Checklist and Explanation. Ann Intern Med. 2018;169:467–473. doi: 10.7326/M18-0850.

For peer review only - http://bmjopen.bmj.com/site/about/guidelines.xhtml
